# Analyzing the association between ferritin levels and ICP using machine learning algorithms: a retrospective case-control study

**DOI:** 10.3389/fmed.2026.1804534

**Published:** 2026-04-17

**Authors:** Na Wei, Shuang Guo, Xiaoya Liu, Ju Zhang, Jianhong Liu, Qian Chen, Wei Dai

**Affiliations:** Department of Obstetrics, Guizhou Provincial People’s Hospital, Guiyang, Guizhou, China

**Keywords:** ferritin, intrahepatic cholestasis of pregnancy, logistic regression, random forest, restricted cubic spline

## Abstract

**Introduction:**

Intrahepatic cholestasis of pregnancy (ICP) is a gestational metabolic disorder characterized by impaired maternal bile acid homeostasis. Emerging evidence suggests that dysregulated iron metabolism may contribute to the pathophysiology of ICP. Nevertheless, the specific alterations in iron metabolism among ICP patients remain unexplored.

**Methods:**

In our retrospective study, we compared serum ferritin levels between normal pregnancies and ICP cases at Guizhou Provincial People’s Hospital from January 2023 to March 2025. Propensity Score Matching, P for trend, restricted cubic spline analysis, and a random forest algorithm were employed to assess the association between serum ferritin and ICP risk.

**Results:**

Elevated serum ferritin concentrations were observed in patients with ICP compared to healthy controls throughout gestation. And it shows a positive correlation with serum hepatic enzyme concentration. PSM analysis identified a significant association between increased ferritin levels in the third trimester and higher ICP risk (AOR = 1.005, 95% CI: 1.001–1.008, *p* = 0.016). RCS modeling revealed a nonlinear relationship: a U-shaped association in the second trimester, with ferritin concentrations between 21.7 and 53.9 ng/mL conferring no significant alteration in risk, and a J-shaped association in the third trimester. The random forest variable importance analysis ranked serum ferritin as the foremost predictor of ICP among all evaluated covariates.

**Discussion:**

In conclusion, our study suggests that increased ferritin levels during pregnancy may increase the risk of ICP, though the underlying mechanisms remain unclear. Future research involving large-sample prospective multicenter cohort studies is warranted to further elucidate these mechanisms.

## Introduction

Intrahepatic cholestasis of pregnancy (ICP) is a maternal liver disorder that occurs specifically in the mid to late stages of pregnancy, characterized primarily by unexplained pruritus and fasting bile acid levels of ≥ 10 μmol/L (1). The principal adverse pregnancy outcome associated with ICP is unexplained intrauterine fetal demise, a risk that persists even in patients receiving bile acid-lowering therapy predominantly based on ursodeoxycholic acid (2). To date, the etiology of ICP remains unclear; however, genetic predisposition, environmental factors, nutrient deficiencies, and gut microbiota dysbiosis (3) have all been implicated in its onset and progression (4). In recent years, accumulating evidence has suggested that patients with ICP exhibit metabolic dysregulation of multiple substances, including lipids, which may play a role in disease progression or in adverse pregnancy outcomes (5). Consequently, evaluating the metabolism of nutritional elements in ICP patients may represent a novel approach for comprehensively understanding the disease and preventing its associated adverse pregnancy outcomes.

Ferritin serves as the primary storage form of iron in the human body, predominantly synthesized in the liver, spleen, and bone marrow (6). Under physiological conditions, ferritin binds free iron in the serum, thereby maintaining iron homeostasis and preventing excessive free iron from inflicting tissue damage (7). Serum ferritin levels remain relatively stable and are minimally influenced by dietary factors, making them a critical indicator for assessing bodily iron reserves (8). Nevertheless, ferritin concentrations are sensitive to inflammatory states; under the influence of inflammatory mediators such as interleukin-6 and tumor necrosis factor-*α*, serum ferritin levels may increase and contribute to the acute phase response (9). Furthermore, the level of free iron in the body significantly impacts serum ferritin concentrations (10). Under normal conditions, human iron metabolism encompasses three primary processes: the absorption of dietary iron in the duodenum, jejunum, and ileum; the synthesis and storage of ferritin in the liver; and the excretion of iron via feces and urine (11).

Recent studies have reported that elevated serum ferritin levels are associated with metabolic dysfunction (12) and fatty liver disease (13). In addition, both low transferrin and high ferritin concentrations have been linked to adverse outcomes in acute liver injury (14). Increased ferritin levels have also been implicated in exacerbating cardiovascular damage in patients with nonalcoholic fatty liver disease (15). Occasional reports have examined the relationship between ferritin and pregnancy-related disorders. For instance, Yang et al. (16) demonstrated in a rat model that the absence of the ferritin light chain disrupts uterine spiral artery remodeling. Moreover, elevated serum ferritin levels have been observed in pregnant women with gestational diabetes, and these levels have been correlated with an increased incidence of macrosomia (17). However, there remains a lack of population-based and mechanistic studies elucidating the metabolic dynamics of ferritin during gestation and its relationship with the development of gestational diabetes or hypertensive disorders of pregnancy.

This study retrospectively examines the characteristics of iron metabolism in pregnant patients diagnosed with intrahepatic cholestasis of pregnancy, aiming to elucidate differences in serum ferritin levels between these patients and women with uncomplicated pregnancies and to clarify the relationship between these parameters. The findings are anticipated to enhance understanding of the metabolic features associated with intrahepatic cholestasis of pregnancy and to inform the development of more robust, evidence-based nutritional care strategies during pregnancy. This case–control study has been reported in line with the STROCSS guidelines (18).

## Materials and methods

### Study design and subjects enrollment

**Figure 1:**
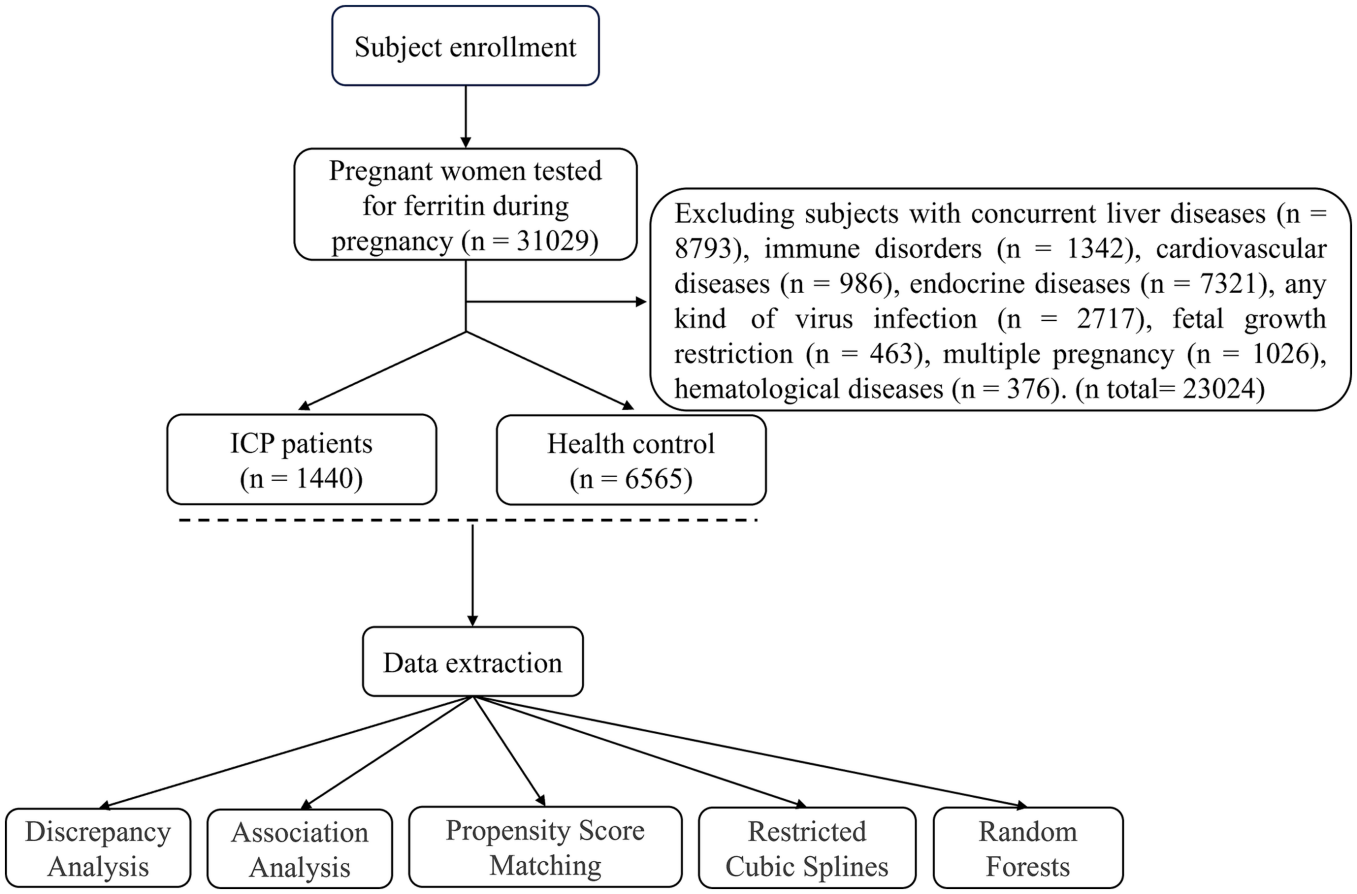
Flowchart illustrating the process of study subjects inclusion.

This study employed a retrospective case–control design to evaluate the association between serum ferritin levels and intrahepatic cholestasis of pregnancy. Subjects presented at Guizhou Provincial People’s Hospital between January 2023 and March 2025 were enrolled. Of an initial cohort of 31,029 participants, 23,024 were excluded based on pre-specified criteria, including pre-pregnancy or gestational diagnosis of hepatitis viral infection, fatty liver disease, biliary stones or polyps, immune system disorders (encompassing autoimmune conditions), cardiovascular diseases, endocrine disorders (including thyroid dysfunction), and hematologic diseases associated with abnormal iron metabolism. Following these exclusions, the final analytical sample consisted of 1,440 patients diagnosed with intrahepatic cholestasis of pregnancy (ICP) and 6,565 individuals with uncomplicated pregnancies (Figure 1).

### Data resource

Demographic, clinical, and laboratory data for all participants were extracted from the electronic medical record and laboratory information systems of Guizhou Provincial People’s Hospital. Retrieved information included maternal age, height, pre-pregnancy weight, gestational weight gain, exposure to hormonal medications during pregnancy, gravidity, and parity, as well as laboratory measurements of alanine aminotransferase (ALT), aspartate aminotransferase (AST), alkaline phosphatase (ALP), total bile acids, and serum ferritin. Serum ferritin levels were categorized into early (10–13 weeks of gestation), mid- (14–27 weeks), and late-pregnancy (28 weeks until delivery) periods according to the gestational week at serum ferritin concentration test. The association between serum ferritin concentration and the risk of ICP was subsequently analyzed within each gestational period.

All data from the full text were forwarded to the corresponding author’s email by data management personnel on July 20, 2025, after the removal of all private information. The data analysis process commenced on 23/07/2025, and concluded on 07/08/2025.

### Diagnostic criteria

In our study, healthy controls were defined as pregnant individuals who did not develop any medical or surgical diseases during pregnancy, passed all prenatal examinations, and ultimately delivered. Intrahepatic cholestasis of pregnancy was diagnosed when patients exhibited an unexplained fasting bile acid level of ≥10 μmol/L or non-fast bile acid level of >19 μmol/L in conjunction with pruritus, in the absence of identifiable precipitating factors and after excluding liver and biliary pathologies, autoimmune diseases, and viral infections (19, 20). Gestational diabetes was defined by the presence of a fasting blood glucose level of ≥5.1 mmol/L, or by the results of an oral glucose tolerance test (OGTT) demonstrating a 1-h blood glucose level of ≥10.0 mmol/L and a 2-h blood glucose level of ≥8.5 mmol/L during pregnancy (21). Gestational anemia was diagnosed when serum hemoglobin levels were below 120 g/dL prior to 13 gestational weeks, and below 110 g/dL from 14 gestational weeks until delivery (22). Iron deficiency during pregnancy was identified by a serum ferritin level of less than 30 ng/mL (23).

### Sample size

The sample size was calculated *a priori* using G*Power software (version 3.1.9.7). For an independent two-sample t-test comparing serum ferritin levels between ICP cases and controls, we assumed a medium effect size (Cohen’s *d* = 0.5) based on prior observational data (24). With a two-sided significance level (*α*) of 0.05 and a statistical power of 80%, the analysis indicated that 64 participants per group were required. Thus, the minimum total sample size needed was 128 participants.

### Statistics

Our study investigates the association between serum ferritin levels and ICP, with serum ferritin serving as the exposure variable and ICP as the outcome variable. Baseline characteristics, including age, body mass index (BMI), gravidity, parity, and gestational weight gain, were compared using a non-paired t-test for normally distributed data, with results expressed as mean ± standard deviation. For variables that did not follow a normal distribution, nonparametric tests were employed, and results are presented as the median (interquartile range). All dichotomous variables, such as the presence of ICP and early pregnancy hormone exposure, were coded as 1 for positive and 0 for negative.

GraphPad Prism (version 8.0) was used to conduct *t*-tests, Mann–Whitney *U* tests, and chi-square tests. Concurrently, R software (version 4.3.1) was employed for multivariate logistic regression analysis using the “glm” package, as well as for propensity score matching and conditional logistic regression via the “matchIt” package (25). Additionally, restricted cubic spline analysis was performed using the “rlm” package, and the random forest algorithm, implemented through the “randomForest” package, was applied to identify the key influencing factors (26).

In our analyses, multivariate logistic regression, propensity score matching, restricted cubic spline analysis, and random forest algorithms were used to explore the association between ICP and serum ferritin concentration. All models adjusted for the following confounding factors: pre-pregnancy body mass index, exogenous hormone exposure during pregnancy, gravidity, parity, gestational weight gain, assisted reproductive technologies, and maternal age.

### Ethical statement

Our study strictly adheres to the Declaration of Helsinki, and the protocol was reviewed and approved by the Ethics Committee of Guizhou Provincial People’s Hospital, with support from the hospital.

## Results

### Comparative analysis of baseline data

Based on our inclusion and exclusion criteria, the study ultimately enrolled 6,565 women with normal pregnancies and 1,440 patients diagnosed with ICP (Figure 1). Initially, we conducted comparative analyses of the subjects’ baseline characteristics, including age, gestational weight gain, gravidity, parity, pre-pregnancy body mass index (BMI), exposure to hormonal medications during pregnancy, and conception via assisted reproductive technologies. The results revealed that patients with ICP were older, had higher gravidity and pre-pregnancy BMI, and were more likely to conceive through assisted reproduction and be exposed to hormonal medications during pregnancy, compared to women with normal pregnancies. Conversely, patients with ICP demonstrated significantly lower gestational weight gain (Table 1).

**Table 1:** Baseline data of subjects.

Variables	Control (*n* = 6,565)	ICP (*n* = 1,440)	*p*-value
Age, year (median, IQR)	30 (5)	31 (5)	0.03
21–29 (%)	2,671 (40.7)	575 (39.9)	
30–34 (%)	3,008 (45.8)	645 (44.8)	
>35 (%)	886 (13.5)	220 (15.2)	
Gravida (median, IQR)	1 (1)	2 (1)	<0.0001
Parity (median, IQR)	0 (0)	0 (0)	<0.0001
GWG (median, IQR)	13 (4.5)	12.5 (5.0)	<0.0001
BMI pre-pregnancy (median, IQR)	20.2 (3.06)	21.32 (3.23)	<0.0001
ART (frequency, ratio)	504 (9.6%)	183 (14.2%)	<0.0001
Progesterone exposure (frequency, ratio)	1,072 (16.3%)	276(19.2%)	<0.0001

GWG, gestational weight gain; BMI, body mass index; ICP, intrahepatic cholestasis of pregnancy; ART, artificial reproductive technology.

### Comparison of differences in serum ferritin concentration

**Figure 2:**
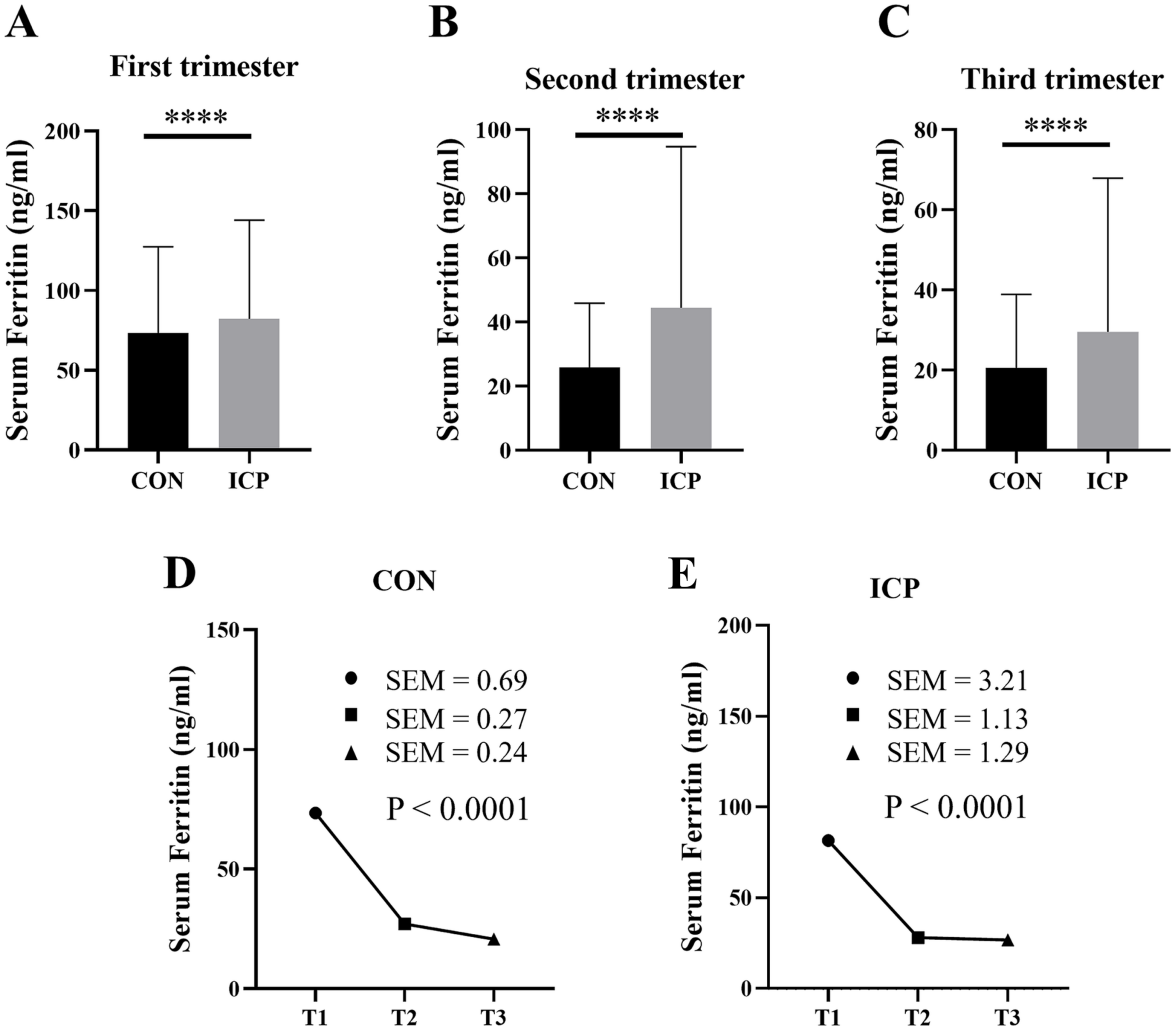
The differences in serum ferritin concentrations and their variations throughout gestation between normally pregnant women and patients with ICP. Panels **(A–C)** compare serum ferritin levels during early, mid, and late gestation, respectively, between the two groups. Panel **(D)** illustrates the temporal changes in serum ferritin concentrations among normally pregnant women, while Panel **(E)** depicts the corresponding fluctuations in ICP patients. Statistical significance is denoted as follows: *p < 0.05, **p < 0.01, ***p < 0.001, and ****p < 0.0001.

Serum ferritin levels were significantly higher in patients with ICP compared to women with normal pregnancies across all trimesters (*p* < 0.05). A longitudinal examination of serum ferritin levels throughout gestation indicated that levels in normal pregnancies gradually declined, reaching a nadir in late pregnancy. In contrast, ICP patients experienced a marked reduction from the first to the second trimester, with a less pronounced decline from mid to late pregnancy relative to the normal cohort (Figure 2). Moreover, comparisons of gestational anemia and iron deficiency between the two groups demonstrated that the incidence of anemia was significantly higher in ICP patients than in normal controls at all stages of gestation. Regarding iron deficiency, no significant differences were observed between the two groups during early pregnancy; however, during mid and late pregnancy, the prevalence of iron deficiency was significantly greater in the normal population than in ICP patients (Table 2).

**Table 2:** Ferritin metabolism and prenatal outcome between two groups.

Variables	CON	ICP	*χ*^2^	*P*-value
Anemia in the first trimester (832)	786/4349	46/160	11.66	<0.001
Anemia in the second trimester (1809)	1308/6499	368/1369	19.28	<0.0001
Anemia in the third trimester (2358)	634/5517	229/1104	50.72	<0.0001
ID in the first trimester (1645)	1168/6565	158/869	0.06	0.81
ID in the second trimester (6563)	4342/6131	432/854	29.39	<0.0001
ID in the third trimester (7681)	5578/6565	983/1342	1.031	0.0012

ID, iron deficiency; ICP, intrahepatic cholestasis of pregnancy.

### Correlation analysis of ferritin levels with TBA and liver enzyme levels in ICP patients

**Figure 3:**
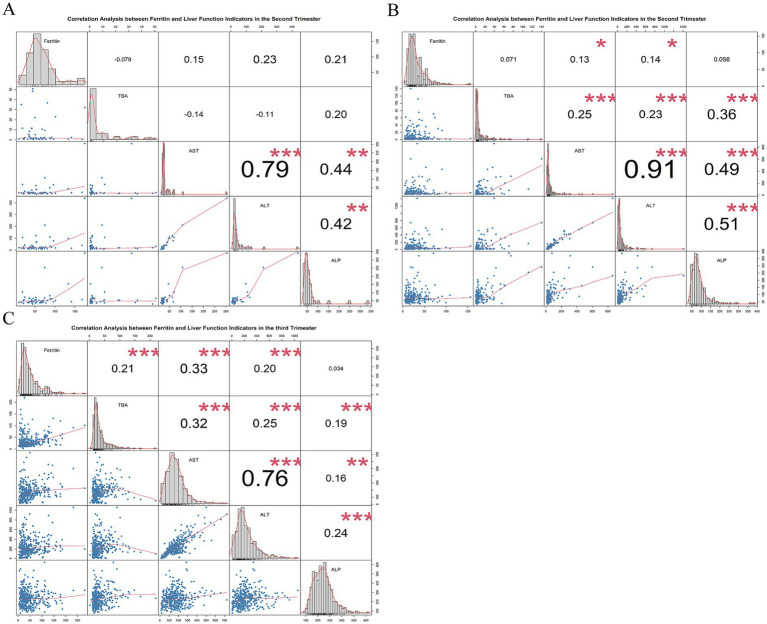
The correlation between serum ferritin concentrations and both liver enzyme and bile acid indices in patients with ICP. **(A)** The correlation analysis during early pregnancy, **(B)** during mid-pregnancy, and **(C)** during late pregnancy. TBA, total bile acids; ALT, alanine aminotransferase; AST, aspartate aminotransferase; and ALP, alkaline phosphatase. Statistical significance is denoted as follows: *p < 0.05; **p < 0.01; ***p < 0.001; ****p < 0.0001.

To investigate the association between serum ferritin and both liver enzymes and total bile acid (TBA) levels in patients with ICP, we conducted a Spearman correlation analysis examining the relationships between serum ferritin and Alanine Aminotransferase (ALT), Aspartate Aminotransferase (AST), Alkaline Phosphatase (ALP), and TBA. In early pregnancy, no significant correlations were observed between serum ferritin levels and the aforementioned liver enzymes or TBA. Conversely, in mid-pregnancy, serum ferritin was significantly positively correlated with ALT (Spearman *r* = 0.13, *p* < 0.05) and AST (Spearman *r* = 0.14, *p* < 0.05). However, in late pregnancy, no significant associations were found between serum ferritin and either liver enzymes or TBA (Figure 3).

### Multivariate logistic regression

**Figure 4:**
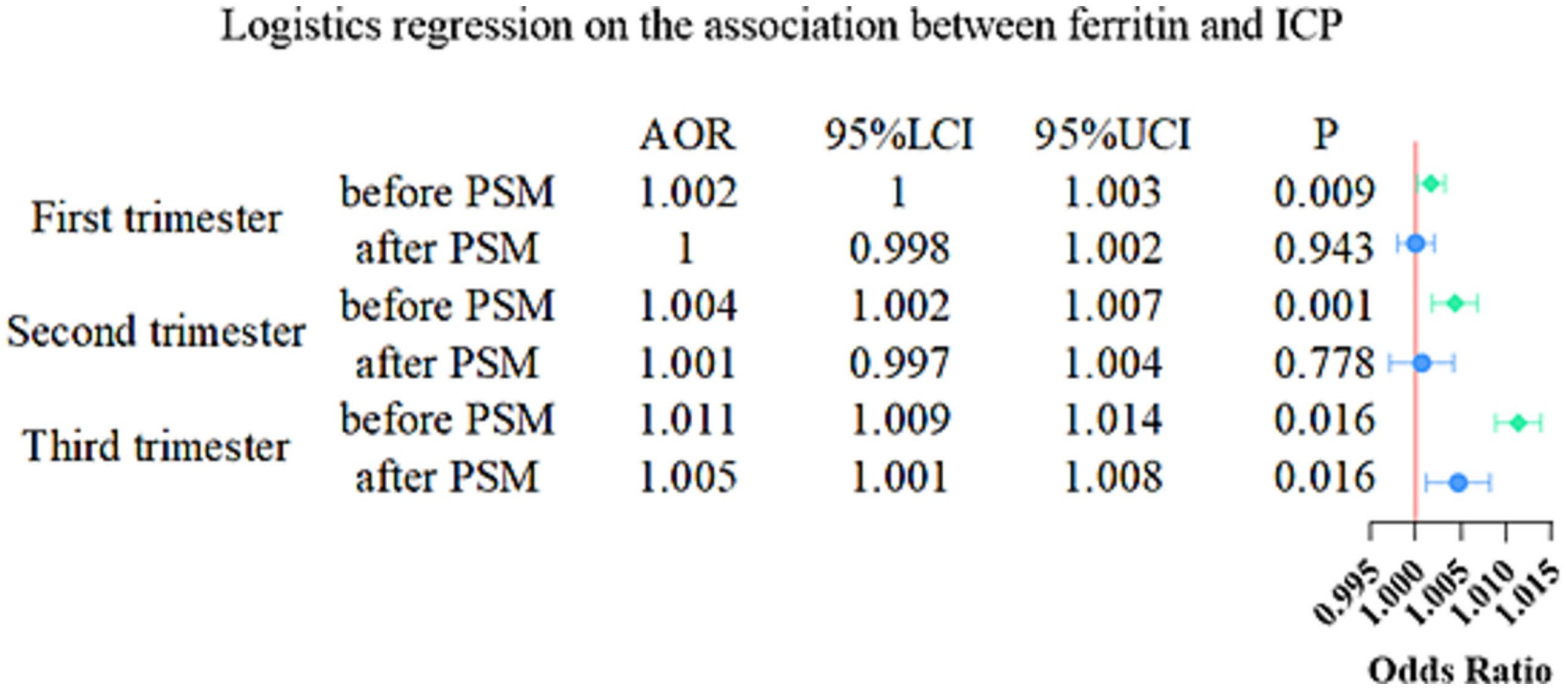
The results of a conditional logistic regression analysis exploring the association between serum ferritin levels and the risk of intrahepatic cholestasis of pregnancy (ICP). PSM, Propensity Score Matching; LCI, Lower confidential interval; UCI, Upper confidential interval.

To examine the association between serum ferritin levels and the risk of ICP, we performed a multivariable logistic regression analysis. The model adjusted for several potential confounders, including pre-pregnancy body mass index, hormonal medication exposure during pregnancy, gravidity, parity, gestational weight gain, use of assisted reproductive technology, and maternal age. The findings indicate that elevated serum ferritin levels in early pregnancy were significantly associated with an increased risk of ICP (AOR = 1.002, 95% CI: 1.000–1.003, *p* = 0.009). Similarly, higher ferritin levels in mid-pregnancy were significantly linked to an elevated risk of ICP (AOR = 1.004, 95% CI: 1.002–1.007, *p* = 0.001), and increased serum ferritin levels in late pregnancy were also significantly correlated with a heightened risk of ICP (AOR = 1.005, 95% CI: 1.001–1.008, *p* = 0.016) (see Figure 4 and Supplementary Figure S1).

### *P* for trend

In light of the association between elevated serum ferritin levels and increased risk of ICP identified in multivariable models across gestation, we performed formal trend analyses to evaluate the presence of a dose–response relationship. These analyses confirmed significant dose–response associations in both the first trimester [per quartile increase: odds ratio (AOR) = 1.07, 95% confidence interval (CI): 1.01–1.14, *P* for trend = 0.03] and the third trimester [per quartile increase: AOR = 1.21, 95% CI: 1.15–1.28, *P* for trend < 0.001] (see Table 3).

**Table 3:** *P* for trend test.

Testing period of pregnancy	*N*	Serum Ferritin (μg/ml, median [range])	AOR (95% CI)	*P* for trend
First trimester	1898	24.60 [1.70–36.10]	Ref.	0.03
	1901	47.30 [36.20–59.90]	0.98 (0.81, 1.19)	
	1894	75.45 [60.00–96.10]	0.93 (0.77, 1.14)	
	1895	131.10 [96.20–562.20]	1.25 (1.04, 1.51)	
	/	Per quartile increase	1.07 (1.01–1.14)	
Second trimester	1813	10.70 [0.40–14.60]	Ref.	0.25
	1785	18.30 [14.70–22.20]	0.77 (0.61, 0.97)	
	1770	27.30 [22.30–35.00]	0.70 (0.55, 0.89)	
	1786	50.80 [35.10–865.80]	0.89 (0.71, 1.12)	
	/	Per quartile increase	0.96 (0.89–1.03)	
Third trimester	2049	8.00 [0.90–11.40]	Ref.	<0.001
	2027	14.70 [11.50–17.70]	0.88 (0.75, 1.05)	
	2043	21.30 [17.80–25.90]	0.91 (0.77, 1.07)	
	2011	33.70 [26.00–557.90]	1.78 (1.52, 2.07)	
	/	Per quartile increase	1.21 (1.15–1.28)	

CI, confidential interval; Ref., references, *P* for trend < 0.05 refers to statistically significant.

### Propensity score matching and conditional logistic regression

To further mitigate the influence of confounding factors and rigorously assess the association between serum ferritin and ICP, we conducted propensity score matching at a 1:1 ratio between ICP patients and normal pregnant women. The bar chart and score distribution plot (Supplementary Figure S2) demonstrate that, following matching, differences in confounding factors (pre-pregnancy body mass index, exogenous hormone exposure during pregnancy, gravidity, parity, gestational weight gain, assisted reproductive technologies, and maternal age) between the two groups were substantially minimized. Subsequently, we performed a conditional logistic regression analysis. The results revealed that after controlling for confounding factors, serum ferritin levels during the first and second trimester were not significantly associated with ICP risk; however, in the third trimester, serum ferritin levels remained significantly associated with ICP risk (AOR = 1.005, 95% CI: 1.001–1.008, *p* = 0.016) (Figure 4 and Supplementary Figure S3).

### Restricted cubic spline analysis

**Figure 5:**
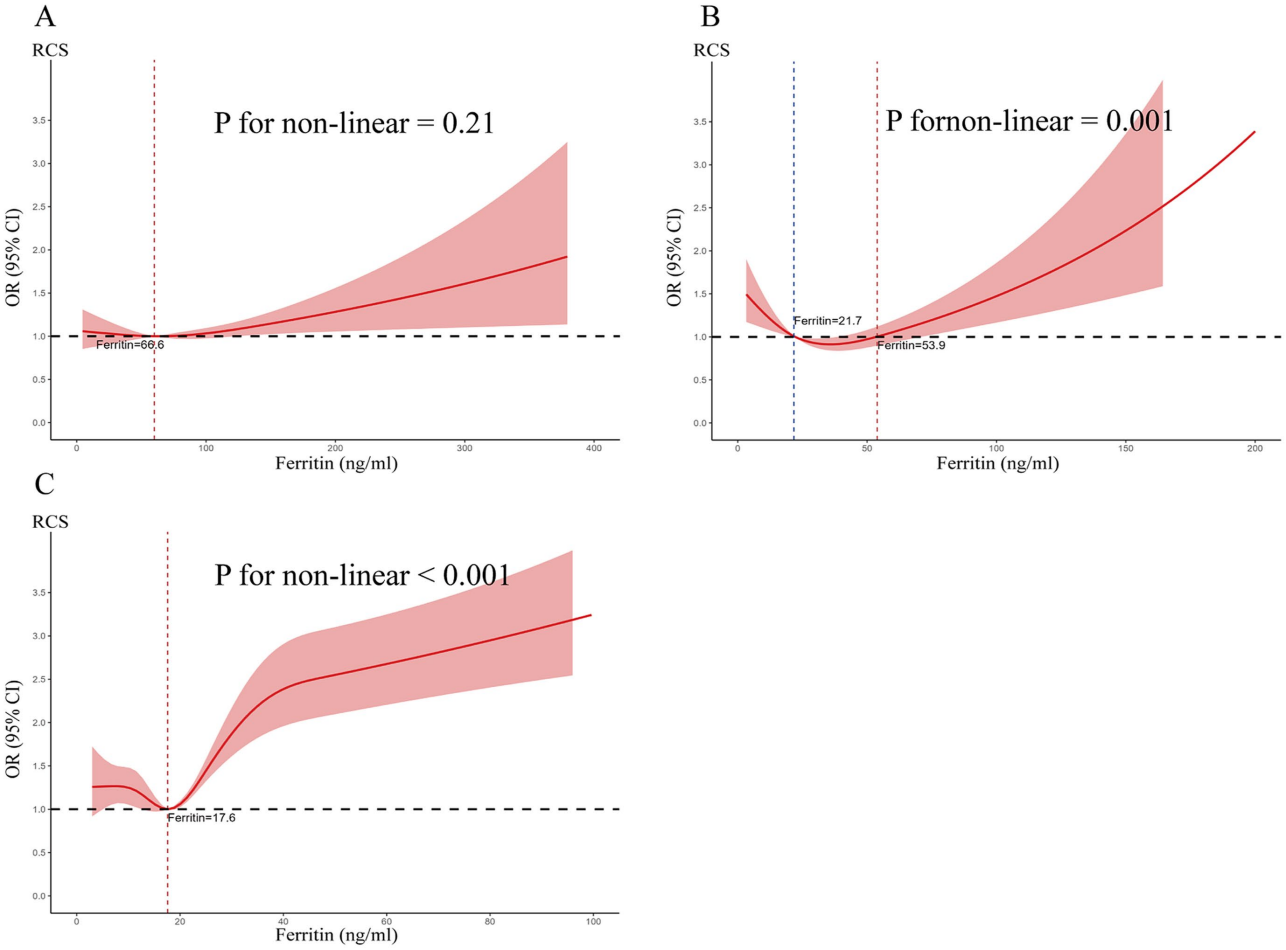
Restricted cubic spline (RCS) analysis evaluating the association between serum ferritin levels during pregnancy and the risk of ICP. **(A)** The RCS analysis of serum ferritin in early pregnancy, 3 notes, (10th, 50th, and 90th percentile, respectively), **(B)** the analysis for mid-pregnancy, 4 notes, (5th, 35th, 65th, and 95th percentile, respectively), and **(C)** the analysis for late pregnancy, 5 notes, (5th, 27.5th, 50th, 72.5th, and 95th percentile, respectively). An odds ratio (OR) of 1.0 was used as the reference for evaluating the risk of ICP.

To further explore the nonlinear relationship between serum ferritin levels and the risk of ICP, we examined the association between serum ferritin levels at various pregnancy stages and the incidence of ICP. Our analysis revealed that, in early pregnancy, serum ferritin levels were not significantly associated with a nonlinear change in ICP risk. However, during mid-pregnancy, a significant U-shaped association emerged. Specifically, our data indicate that the lowest risk of ICP is observed when serum ferritin levels range between 21.7 and 53.9 ng/mL. In late pregnancy, a J-shaped nonlinear relationship was identified, with an inflection point at a serum ferritin level of 17.6 ng/mL (Figure 5).

### Random forest algorithm

**Figure 6:**
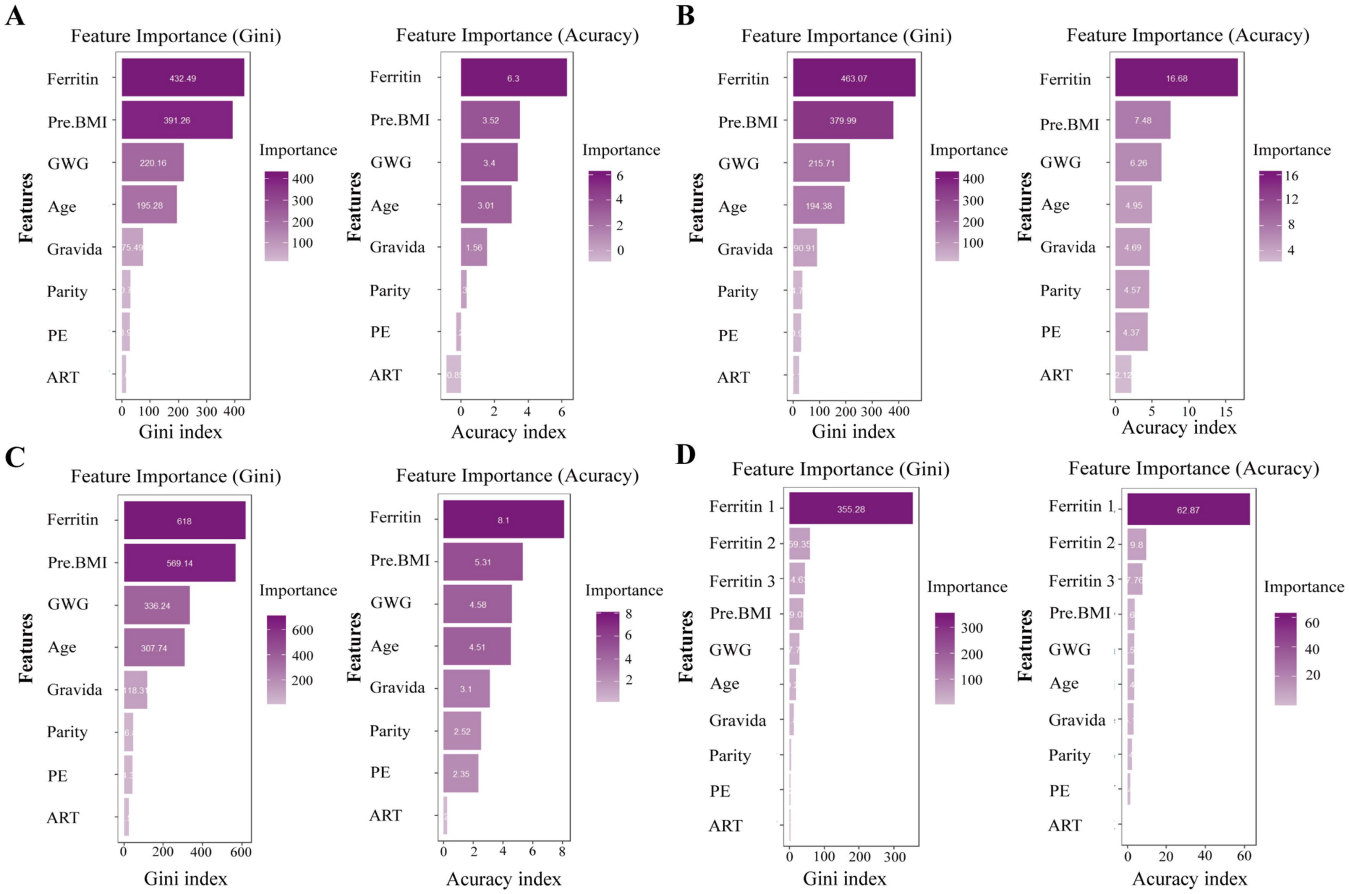
Analysis of risk factors for ICP using the random forest algorithm. Panel **(A)** presents the analysis for early pregnancy, panel **(B)** for mid-pregnancy, panel **(C)** for late pregnancy, and panel **(D)** for the entire gestational period. Ferritin1, Ferritin2, and Ferritin3 indicate ferritin concentrations in early, mid, and late pregnancy, respectively. The abbreviations are defined as follows: Pre. BMI, pre-pregnancy body mass index; PE, exposure to hormonal medications during pregnancy; GWG, gestational weight gain; DGA, gestational age at detection; and ART, assisted reproductive technology.

To further investigate the association between serum ferritin levels and ICP, we assessed the relative importance of various factors using the random forest algorithm (confusion matrices see in Supplementary Figure S4). Our analysis revealed that, regardless of whether measurements were taken during the first, second and third trimesters, serum ferritin consistently emerged as the most significant predictor of ICP onset compared to other risk factors, such as exposure to pregnancy hormones and assisted reproductive conception. To identify the gestational period during which serum ferritin most strongly influences ICP risk, we performed factor importance rankings on a cohort of patients tested in all three trimesters. Both the Gini index and precision methods indicated that serum ferritin measured in early pregnancy held greater importance than in mid-pregnancy, which in turn was more influential than measurements taken in late pregnancy (Figure 6).

## Discussion

To our knowledge, the present study is the first to elucidate an association between serum ferritin and ICP. In this study, we investigated the association between serum ferritin levels and ICP and evaluated the impact of several contributing factors by analyzing data from 6,565 women with normal pregnancies and 1,440 cases of ICP. To explore the associations between serum ferritin concentration and ICP, we employed a range of statistical techniques, including multivariate logistic regression, propensity score matching, restricted cubic splines, and random forest algorithms. Our data indicate that serum ferritin concentrations were consistently elevated in patients with ICP compared to healthy controls throughout gestation. While multivariable logistic regression identified a significant association between serum ferritin levels and ICP risk across all pregnancy periods, propensity score-matched analysis revealed that this association remained statistically significant only for ferritin levels measured during the third trimester. Our findings also indicate that serum ferritin levels in the third trimester are significantly associated with an increased risk of ICP which shows a dose–response relationship. Furthermore, among all covariates (maternal age, pre-pregnant weight, gravidity, parity, exogenous hormone exposure during pregnancy, and weight gain during pregnancy) included in the variable importance analysis, serum ferritin level was ranked as the most significant predictor in all three gestational periods in the random forest analysis.

Previous studies have extensively documented an association between serum ferritin levels and the risk of various pregnancy-related disorders (27). Fang et al. (28) conducted a retrospective analysis of data from more than 30,000 individuals in Fujian, China, including 1,103 patients diagnosed with hypertensive disorders of pregnancy (HDP). Their findings demonstrated that early pregnancy serum ferritin levels were significantly higher in patients with gestational hypertension compared to those in normotensive pregnancies. Furthermore, a subsequent random forest analysis indicated that serum ferritin levels in early pregnancy were more effective in predicting HDP than levels measured during mid-to-late pregnancy (28). Similarly, Xie et al. (29) reported that patients with gestational diabetes mellitus (GDM) exhibited elevated serum ferritin levels relative to women with normal pregnancies and that these elevated levels were associated with an increased risk of developing GDM. In this present study, we report a positive association between serum ferritin levels and ICP, which is consistent with the associations previously published.

Currently, ferritin is primarily recognized as the principal marker of iron storage, with reduced serum ferritin levels employed to predict and diagnose iron deficiency anemia (8, 30). However, as previously noted, ferritin also serves as a relatively sensitive indicator of the body’s inflammatory status. It is widely acknowledged that during normal physiological pregnancy, maternal inflammatory levels are comparatively high in the first trimester to facilitate embryo implantation, decrease slightly in the second trimester to support gestational maintenance, and then rise again in the third trimester, particularly near delivery (31). Nonetheless, studies have demonstrated that excessively elevated inflammatory levels are implicated in the pathogenesis of various pregnancy-related conditions, such as GDM (32), preeclampsia (33), and preterm birth (34). Consequently, the causal relationship between serum ferritin levels and disease risk, as observed in numerous studies, remains ambiguous. In our study, although the overall incidence of anemia in patients with ICP was higher than that in women with normal pregnancies, the prevalence of iron deficiency in the second and third trimesters were lower in ICP patients compared with their normal counterparts. This finding suggests that, in the context of ICP, relying solely on serum ferritin levels to assess iron metabolism and diagnose iron deficiency anemia may be unreliable, and the association between ICP and serum ferritin levels need to be further proved.

Our findings still demonstrated that, in the second and third trimesters, serum ferritin levels of patients with ICP are significantly and positively correlated with liver enzyme indices, specifically ALT and AST. This observation suggests that elevated serum ferritin levels may play a role in impairing liver function during the course of the disease. A similar association was reported in a study on hepatitis C virus–infected patients, which demonstrated a significant correlation between ferritin and liver enzyme levels (35). Furthermore, research conducted by Hao et al. (36) reported that increased ferritin concentrations in pediatric patients with infection-related hemophagocytic lymphohistiocytosis were significantly linked to liver dysfunction. However, the precise mechanisms underlying the relationship between elevated serum ferritin levels and liver damage remain to be fully elucidated.

Previous investigations have proposed that high ferritin levels might promote the infiltration of neutrophils into hepatic tissue and the formation of neutrophil extracellular traps (NETs), thereby exacerbating hepatocellular injury (37). Additionally, other studies have reported that elevated ferritin levels and increased transferrin saturation may activate NF-κB mediated inducible nitric oxide synthase, leading to iron accumulation and oxidative inflammation, which in turn could induce ferroptosis and disrupt estradiol biosynthesis in the ovaries of aged rats (38–40). In addition, a previous study demonstrated that reduced levels of light-chain ferritin at the placental site in patients with ICP induce ferroptosis (41). Of note, this phenomenon has also been observed in patients with hypertensive disorders of pregnancy (16). Tang et al. (42) reported that elevated expression of ACADVL at the placental site in patients with ICP is associated with lipid oxidative metabolism disorders, resulting in increased DRP1 levels, reduced MFN2 and GPX4 levels, and elevated ROS and MDA levels in the intracellular circulation, thus participating in the pathological progression of ICP. Thus, we hypothesize that increased ferritin levels might drive ICP pathogenesis only via liver function impairment, which should be examined in future research.

Interestingly, our RCS analysis revealed that excessively low serum ferritin levels during the second and third trimesters were also associated with an increased risk of ICP. Nevertheless, to the best of our knowledge, no previous study has reported this phenomenon. This finding may be attributable to the characteristics of the study population. Accordingly, our results suggest that maintaining serum ferritin levels within an optimal range during pregnancy may be beneficial for maternal health. The specific thresholds defining this range warrant further investigation through large-scale clinical studies.

In summary, this study is the first to report elevated serum ferritin levels in patients with ICP compared to healthy pregnant controls, with levels showing a significant positive correlation with hepatocellular injury markers (ALT and AST), and to identify a specific association between increased ferritin and higher ICP risk. Maintaining serum ferritin levels within the range of 21.7–53.9 ng/mL during the second trimester may be associated with a reduced risk of developing ICP. However, several limitations must be noted: the retrospective design precluded analysis of inflammatory markers; key iron metabolism indicators and iron supplementation during pregnancy were unavailable; and the findings demonstrate association rather than causation. Consequently, future research should employ larger, multicenter prospective cohorts to validate these observations and incorporate controlled mechanistic studies to elucidate the precise role of ferritin in ICP pathogenesis.

## Conclusion

Our study is the first to explore an association between serum ferritin levels during pregnancy and the risk of ICP. Although our results indicate a significant correlation between elevated serum ferritin levels and an increased risk of ICP, these findings should be interpreted with caution. Future research integrating large-scale, multicenter clinical investigations with fundamental studies is essential to elucidate the underlying mechanisms of this association.

## Data Availability

The original contributions presented in the study are included in the article/Supplementary material, further inquiries can be directed to the corresponding author.
