# Supplementary figures and images for: Analyzing the association between ferritin levels and ICP using machine learning algorithms: a retrospective case-control study
Source: Front Med (Lausanne). 2026 Apr 17;13:1804534. doi: 10.3389/fmed.2026.1804534 (PMC13133004; doi:10.3389/fmed.2026.1804534)

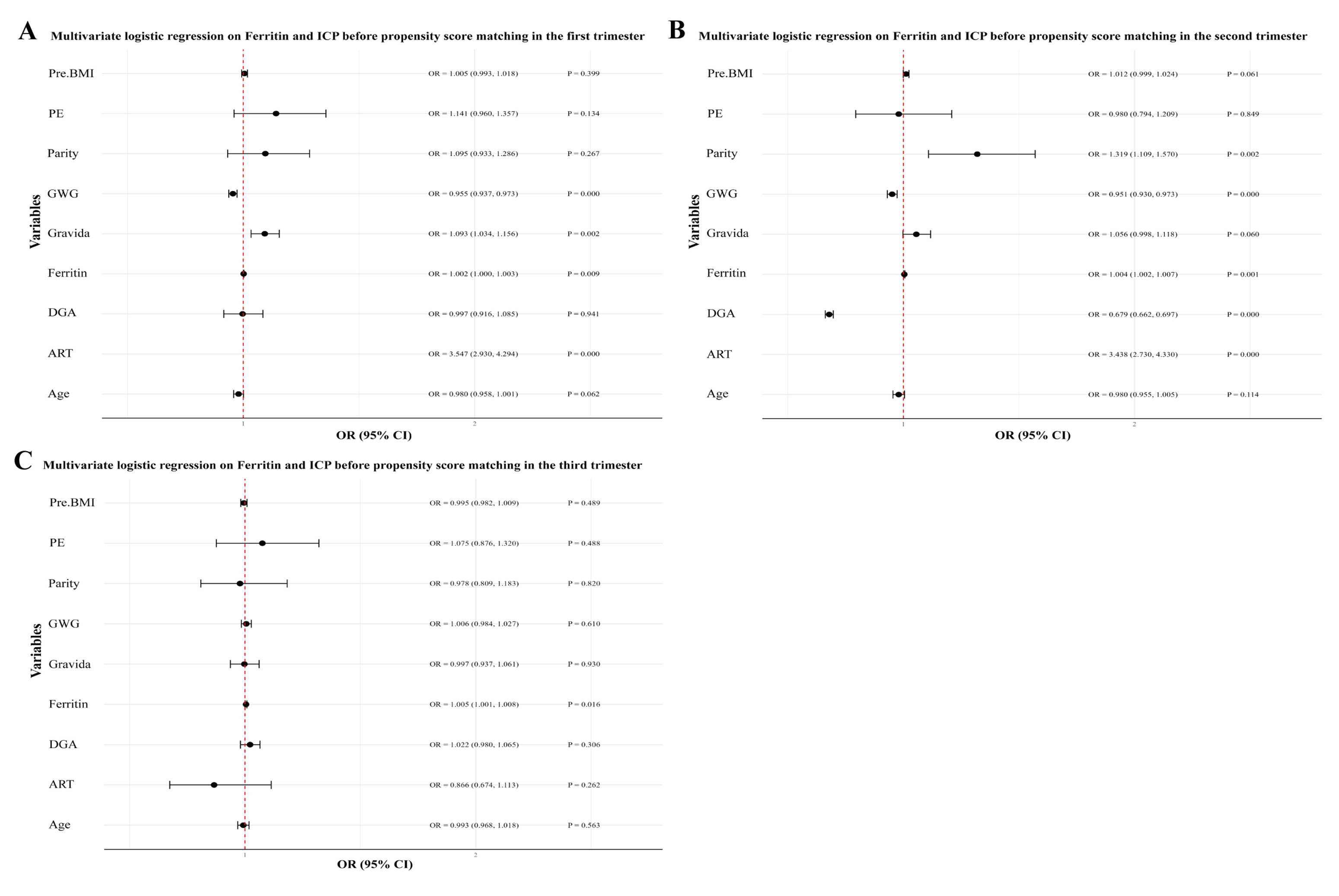

Supplement: Supplementary file 2 [file Image_1.TIF]

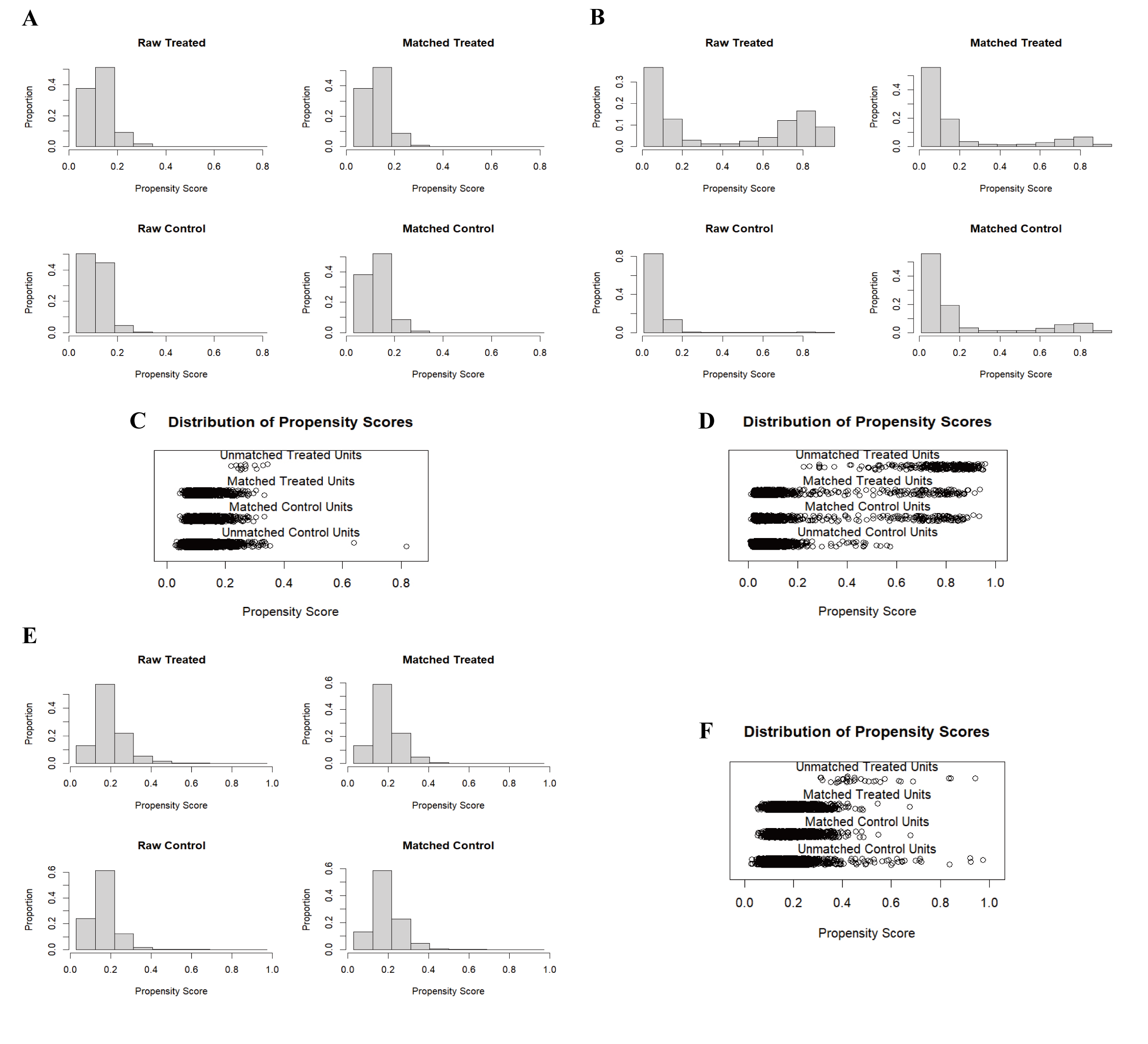

Supplement: Supplementary file 3 [file Image_2.TIF]

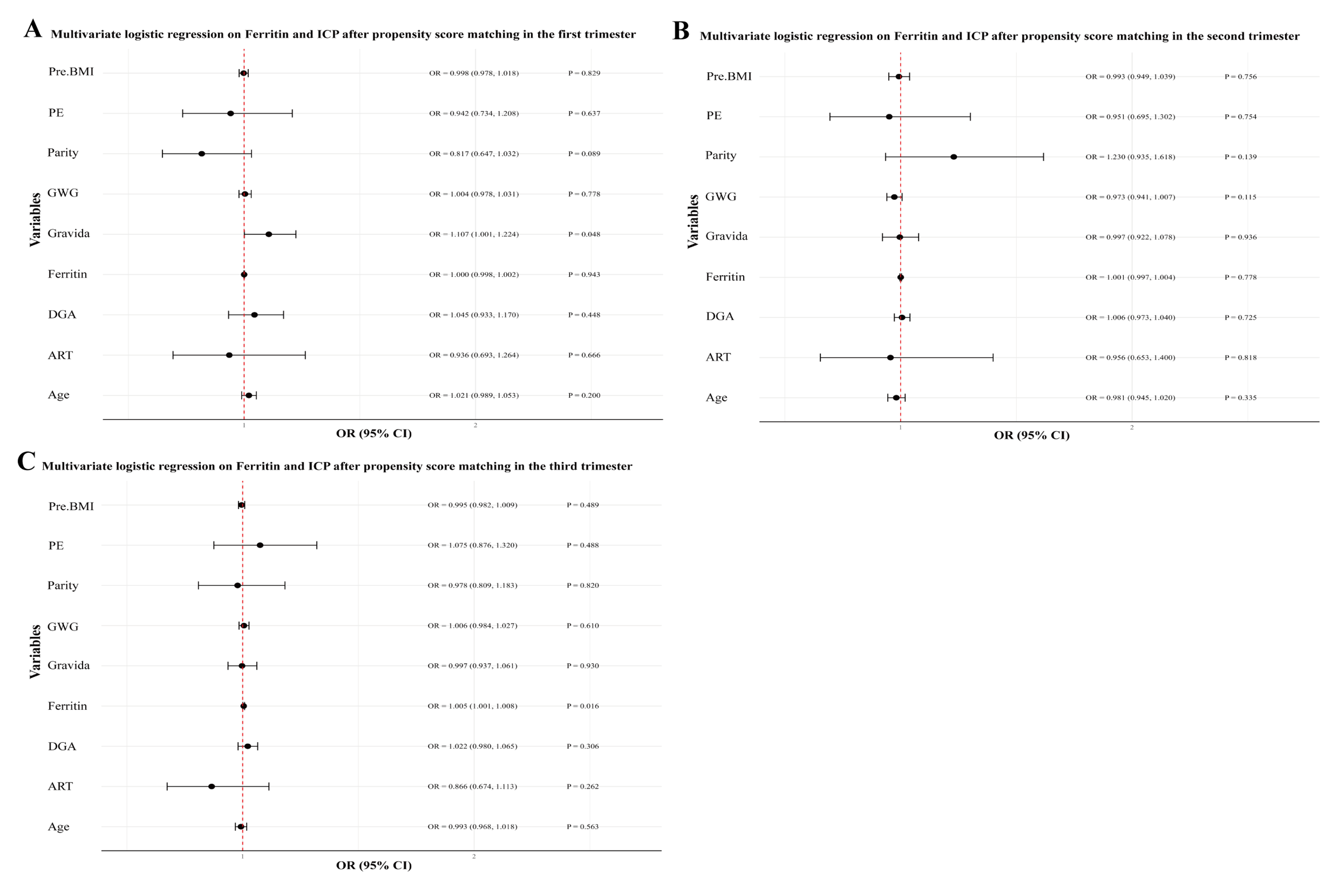

Supplement: Supplementary file 4 [file Image_3.TIF]

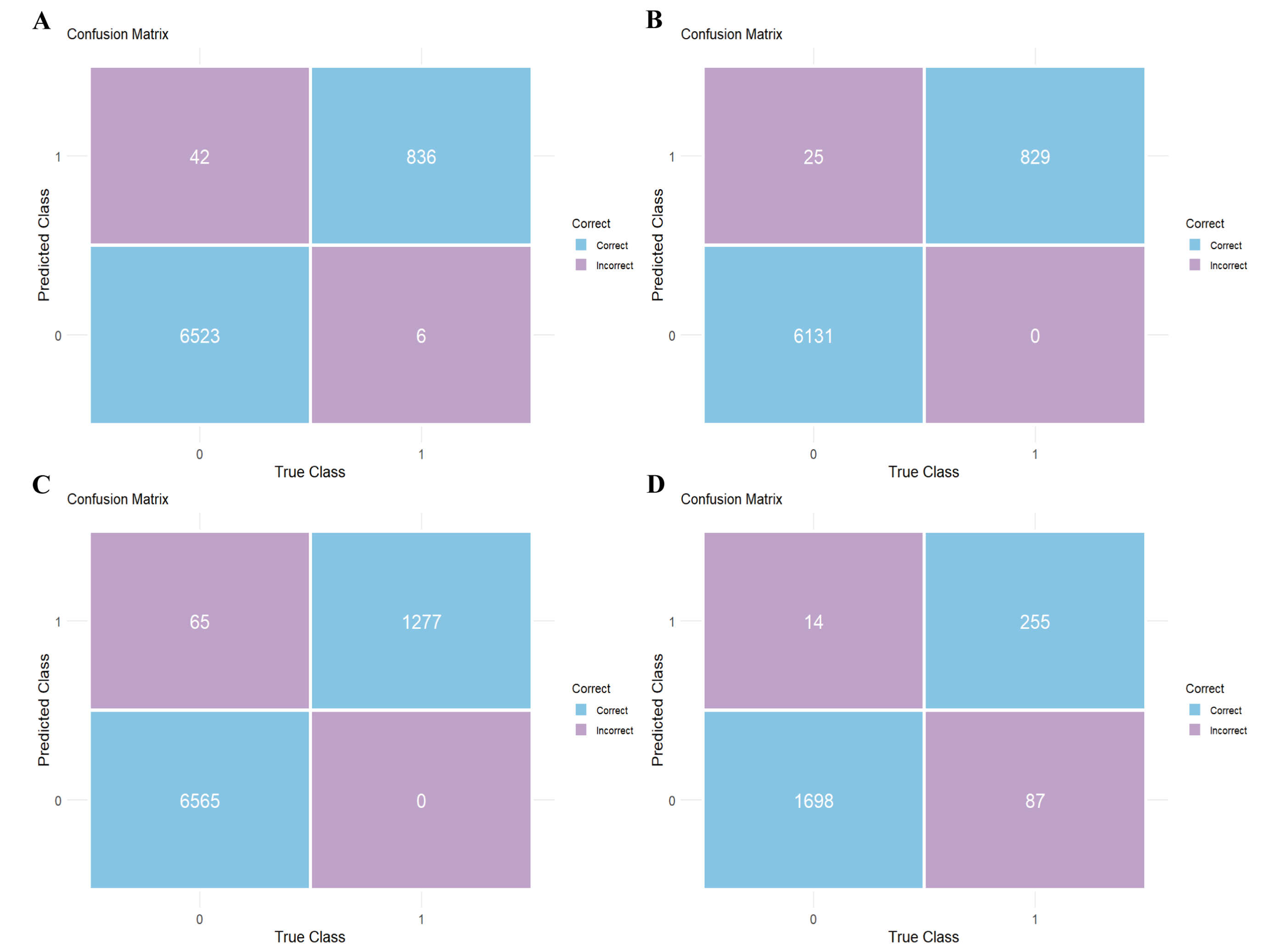

Supplement: Supplementary file 5 [file Image_4.TIF]
