# Supplementary material for: Analyzing the association between ferritin levels and ICP using machine learning algorithms: a retrospective case-control study
Source: Front Med (Lausanne). 2026 Apr 17;13:1804534. doi: 10.3389/fmed.2026.1804534 (PMC13133004; doi:10.3389/fmed.2026.1804534)
Supplement: Supplementary file 1 [file Data_Sheet_1.PDF]

Dear Editor,

Thank you for your letter and for giving us an opportunity to revise our manuscript. We greatly appreciate you and the reviewers' constructive comments and suggestions on our manuscript entitled as "Analyzing the Association Between Ferritin Levels and ICP Using Machine Learning Algorithms: A Retrospective Case-Control Study" (Manuscript ID: 1804534). Those comments are all valuable and very helpful for revising and improving our paper, as well as the important guiding significance to our researches. We have studied the reviewer's comments carefully on the manuscript and made corrections which we hope meet with approval. The main corrections are in the manuscript and the responds to the reviewers' comments are followed below.

We appreciate your assistance in this matter and are glad to respond to any further questions and comments that you may have.

Sincerely yours,

Wei Dai, Ph.D.
Corresponse author:
Wei Dai, Ph.D. Professor, Department of Obstetrics, Department of Obstetrics, Guizhou Provincial People's Hospital, No. 83 Zhongshan East Road, Nanming District, Guiyang City, Guizhou Province, China. E-mail: 18886008855@163.com.

Responses to the reviewers' comments.

Reviewer #1 (Comments for the Author):

Thank you for your insightful and professional review. Your comments have not only guided the revision of our manuscript but also illuminated the direction of our future research. We provide below our responses to your valuable suggestions and the revisions made accordingly.

Q1. Lines 83-85, 114-115

The authors excluded hematologic diseases associated with abnormal iron metabolism, however, it is unclear whether pregnant women receiving iron supplementation were excluded or adjusted for in the analysis. Since serum ferritin is strongly influenced by iron supplementation during pregnancy, this factor may confound the observed association between ferritin levels and ICP risk. The authors should clarify whether iron supplementation data were available and considered.

Responding: Thank you for your insightful and constructive comments. This study is a retrospective case-control study. We acknowledge that the lack of dietary data constitutes a limitation, which has been disclosed in the final paragraph of the Discussion section (subsection on study limitations). Patients with iron metabolism disorders may present with marked abnormalities in ferritin levels, which could affect the final statistical results. Therefore, our study was designed to exclude individuals with iron metabolism disorders. In addition, due to the absence of data on iron supplementation, we were unable to analyze dietary iron intake (line 314-317). The findings reported here represent interim results of a data analysis. In future studies, our team plans to adopt prospective designs to further investigate the associations between iron supplementation, ferritin levels, and ICP. Once again, we sincerely appreciate your professional advice. Your rigorous and meticulous scientific spirit serves as an exemplary model for early-career researchers like us.

Q2. Lines 283-300

Although ferritin is discussed as an inflammatory marker, the manuscript does not adequately explain how elevated ferritin may contribute to ICP pathogenesis, which primarily involves bile acid metabolism dysfunction. The discussion could be strengthened by addressing potential mechanisms such as oxidative stress, ferroptosis pathways, or bile acid transporter dysfunction (e.g., BSEP).

Responding: We greatly appreciate your constructive comments, which have been instrumental in improving the quality of our manuscript. In accordance with your suggestion, we have now included in the Discussion section that elevated ferritin levels may participate in the pathogenesis of ICP by

inducing inflammatory responses and promoting ferroptosis in hepatocytes, consequently leading to hepatic dysfunction. The corresponding revisions have been made in the Discussion section (line 296-301). We thank you once again for your valuable input.

Q3. Lines 218-221

The RCS analysis identified a U-shaped association between ferritin levels and ICP risk during mid-pregnancy. However, the discussion focuses mainly on elevated ferritin levels and does not address the potential implications of low ferritin levels. The authors may consider discussing whether iron deficiency could also contribute to ICP risk.

Responding: Current obstetric perinatal care primarily focuses on iron supplementation, often neglecting the potential risks associated with ferritin overload. Excessive iron supplementation may therefore exert unintended adverse effects. Consequently, to emphasize the potential detrimental effects of iron supplementation, we had initially omitted a discussion of low ferritin levels. In accordance with your valuable guidance, we have now incorporated the relevant discussion to prevent any misunderstanding of our findings (line 304-310). We sincerely appreciate your constructive comments, which have greatly enhanced the quality of our study**.**

Q4. Lines 94-98

Ferritin levels were categorized into early, mid, and late pregnancy. However, it is unclear whether all participants had ferritin measurements in each trimester. Although the manuscript appears to include a subgroup with ferritin measured across all three gestational stages, the sample size of this subgroup is not clearly reported, nor is it explained how missing ferritin measurements were handled in the analysis.

Responding: We appreciate your insightful question. In our study cohort, a subset of patients underwent serum ferritin testing in all three trimesters of pregnancy. In order to comprehensively assess the impact of ferritin levels—particularly to account for temporal dynamics and to evaluate gestational week-specific effects with a larger sample size—we retained all available samples from the three trimesters. Moreover, in the final random forest analysis for variable importance, we specifically compared patients who had ferritin measurements available across all three trimesters.

Q5. Figure 1 reports that 23,024 participants were excluded, but the number excluded for each criterion is not specified. To improve transparency and align with STROBE/STROCSS reporting standards, the authors should provide the number of participants excluded for each condition.

Responding: We are grateful for your constructive suggestion. We sincerely regret this limitation in our study. In accordance with your guidance, we have modified the sample exclusion process described in Figure 1 of the manuscript. We thank you once again for your valuable advice, which

has rendered our work more scientifically sound and methodologically rigorous.

Q6. The methodological description for the RCS analysis in Figure 5 is incomplete. Although P-values for non-linearity are displayed within the figure panels, they are not explicitly reported or discussed in the Results section of the text. Furthermore, to ensure transparency and reproducibility, the authors should specify the number and location of knots (e.g., at which percentiles) used in the models. It is also recommended to define the reference value used for the Odds Ratio (OR = 1.0) for each trimester to clarify the clinical interpretation of the U-shaped and J-shaped curves.

Responding: In this study, the number of knots for the restricted cubic spline (RCS) analysis was selected based on the minimum Akaike information criterion (AIC) value. For the first trimester, the AIC values were 3944.557 for 3 knots, 3501.546 for 4 knots, and 3503.283 for 5 knots. Therefore, three knots were selected, located at the 10th, 50th, and 90th percentiles, respectively. For the second trimester, the AIC values were 2348.017 for 3 knots, 2348.012 for 4 knots, and 2349.947 for 5 knots. Accordingly, four knots were selected, placed at the 5th, 35th, 65th, and 95th percentiles, respectively. For the third trimester, the AIC values were 3943.773 for 3 knots, 3934.35 for 4 knots, and 3931.767 for 5 knots. Consequently, five knots were selected, located at the 5th, 27.5th, 50th, 72.5th, and 95th percentiles, respectively. The reference value for the odds ratio is 1.0. We have incorporated the corresponding content into the legend of Figure 5 (line 470-473).

Q7. The random forest results shown in Figure 6 present variable importance rankings; however, the manuscript does not report model performance metrics such as AUC, sensitivity, specificity, or accuracy. Providing these metrics would help assess the predictive performance and clinical utility of the model.

Responding: Thank you for your insightful suggestion. In this study, our primary aim was to investigate the impact of serum ferritin levels during pregnancy on ICP. To this end, we employed the random forest algorithm for variable importance assessment. In this algorithm, our main objective was to compare the importance of variables, and we selected well-established independent risk factors for ICP, such as hormone exposure during pregnancy and BMI[1] for comparison. Our results indicated that the variable importance of ferritin levels in relation to ICP was even higher than that of hormone exposure during pregnancy. However, the R^2 value of our model was relatively low, which may be attributable to the retrospective study design and incomplete inclusion of relevant variables. To avoid overuse and overinterpretation of statistical methods and tools, we did not perform further analyses such as area under the curve (AUC) in this study.

Reviewer #2 (Comments for the Author):

We are sincerely grateful for the time and expertise you have devoted to reviewing our study, and we greatly appreciate your recognition of our work. Your rigorous and

meticulous scholarly attitude sets a lifelong benchmark for us as researchers. Presented below are our responses to each of your insightful comments, together with the revisions made accordingly, which we sincerely hope will satisfy your expectations.

Q1 (1) Please edit figure 5. Spacing is needed in sub-part B between for and non-linear.

Responding: We are grateful for your meticulous review and constructive comments. We sincerely regret the technical oversights in our manuscript. The revised manuscript has been amended accordingly, and we hope that our revisions will satisfy your expectations. We thank you once again for your invaluable suggestions.

Reference

[1]. Gao XX YM, Liu Y, Li JY, Li L, Chen W, Lu X, Nie G, Chen YH. Prevalence and risk factors of intrahepatic cholestasis of pregnancy in a Chinese population [J]. Sci Rep. 2020;10(1):16307.
